# Novel approach for label free super-resolution imaging in far field

**DOI:** 10.1038/srep13274

**Published:** 2015-09-03

**Authors:** Sergey A. Alexandrov, James McGrath, Hrebesh Subhash, Francesca Boccafoschi, Cinzia Giannini, Martin Leahy

**Affiliations:** 1 Tissue Optics & Microcirculation Imaging Group, School of Physics, National University of Ireland, Galway, Ireland; 2 Department of Health Sciences, University of Piemonte Orientale “A. Avogadro”, 28100 Novara, Italy; 3 Institute of Crystallography, National Research Council, via Amendola 122/O, Bari 70126 Italy

## Abstract

Progress in the emerging areas of science and technology, such as bio- and nano-technologies, depends on development of corresponding techniques for imaging and probing the structures with high resolution. Recently, the far field diffraction resolution limit in the optical range has been circumvented and different methods of super-resolution optical microscopy have been developed. The importance of this breakthrough achievement has been recognized by Nobel Prize for Chemistry in 2014. However, the fluorescence based super-resolution techniques only function with fluorescent molecules (most of which are toxic and can destroy or lead to artificial results in living biological objects) and suffer from photobleaching. Here we show a new way to break the diffraction resolution limit, which is based on nano-sensitivity to internal structure. Instead of conventional image formation as 2D intensity distribution, in our approach images are formed as a result of comparison of the axial spatial frequency profiles, reconstructed for each image point. The proposed approach dramatically increases the lateral resolution even in presence of noise and allows objects to be imaged in their natural state, without any labels.

Most of the fundamental pathological processes in living tissues, such as cancer, exhibit changes at the nanolevel. Existing high resolution microscopy techniques, including near field imaging (Near Field Scanning Optical Microscopy (NSOM or SNOM)) which breaks the resolution limit by exploiting the properties of evanescent waves^1^,^2^,^3^, electron and atomic force microscopy^4^, impose strong limitations on the imaged sample and are unsuitable for the study of live biomedical objects. The best modality for realization of the super-resolution imaging in optical range in far zone is fluorescence microscopy, where the sample acts as a light source itself, providing a very high signal-to-noise (SNR) ratio. Different super-resolution microscopy techniques using fluorescent molecules have been proposed^5^,^6^,^7^,^8^,^9^,^10^,^11^,^12^,^13^,^14^, but all these techniques are based on intrinsic marker properties and require labeling which limits their ability for imaging of living objects *in vivo*.

Different techniques for label free super-resolution imaging have been also proposed, including synthetic aperture microscopy^15^,^16^,^17^, optical nanoscopy using optically transparent microspheres as far-field superlenses (FSL)^18^,^19^, methods based on the use of a special optical mask to remove the need for evanescent fields^20^, coherent total internal reflection dark-field microscopy^21^, far-field vibrational infrared (IR) absorption microscopy^22^, image scanning microscopy which uses point scanning in combination with wide field detection^23^. Most of published label free super-resolution methods permit extended resolution, but resolution is still limited to a finite value and theoretically unlimited resolution cannot be achieved^5^. Existing techniques are complicated, expensive, and hardly can be used for *in vivo* imaging of live objects. In spite of numerous efforts and great achievements in super-resolution microscopy, the challenge now is to make high resolution imaging more accessible and more usable *in vitro* and *in vivo*. There remains a great need for further development and creation of new alternative approaches for label-free super-resolution imaging for investigation of biological objects in their natural environment.

A new approach to probe three-dimensional (3D) structure in far field at nanoscale, based on spectral encoding of spatial frequency (SESF), has been developed recently^24^,^25^,^26^,^27^. Transmittance of instantaneous lateral spatial frequency bandwidth wider than the optical system’s diffraction limit through a low numerical aperture (NA) optical system^24^, and high resolution imaging^25^, based on spectral encoding of the lateral spatial frequency, have been demonstrated. The ability to reconstruct the axial (along depth) spatial frequency profiles for each point of the image with nano-scale sensitivity to structural changes has been shown^26^ and adaptation of the SESF approach for depth resolving imaging has been published^27^,^28^. Here we report a novel approach, based on spectral encoding of the axial spatial frequency, to break the diffraction limit in far zone and dramatically improve resolution in the lateral direction.

## Results

### Numerical simulation

**Figure 1:**
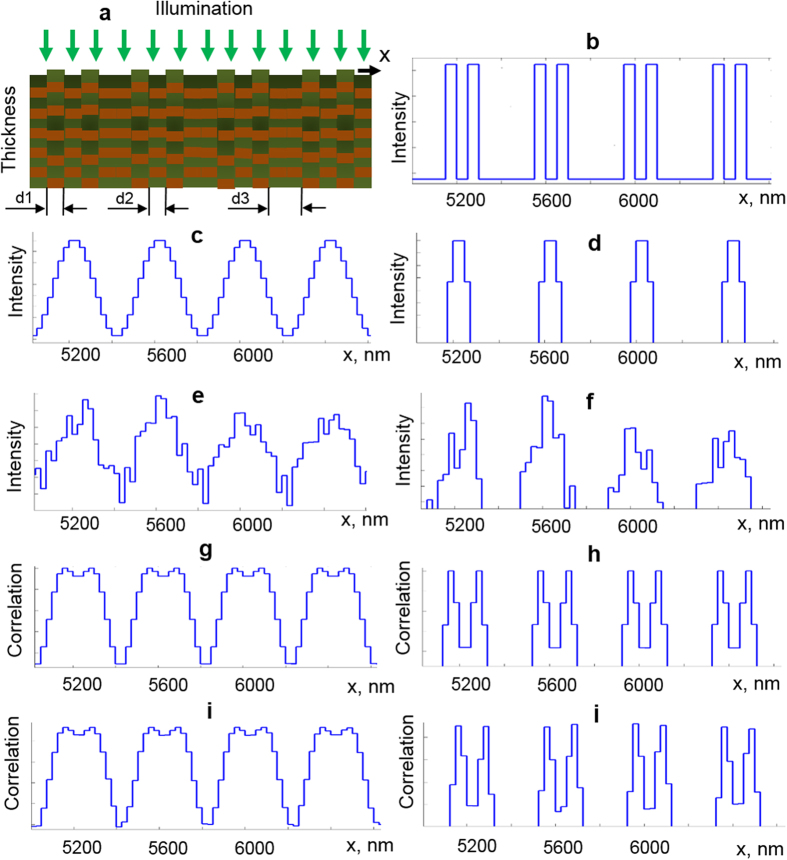
Results of numerical simulation. (**a**) Simulated object, *d1* = 50 nm, *d2* = 50 nm, d3 = 250 nm. (**b**) Lateral intensity distribution of the reflected light from the object before convolution. (**c–f**), Intensity distributions in image plane using conventional microscopy. (**g–j**) Correlation coefficient distributions in image plane using srSESF approach. (**c**,**d**,**g**,**h**)– without noise; (**e**,**f**,**i**,**j**)—with noise. (**d**,**f**,**h**,**j**) are magnified portions of (**c**,**e**,**g**,**i**).

To validate the novel super-resolution SESF (srSESF) approach we performed numerical simulation of the imaging process (see METHODS for details). A sample, which consists of pairs of lateral areas, width *d*_*1*_, with similar axial structure separated by area *d*_*2*_with different axial structure is illuminated by broadband plane wave. Reconstruction of the initial intensity profile (Fig. 1b) immediately after reflection from the sample (Fig. 1a) via conventional microscopy is presented in Fig. 1c–f. These results demonstrate that the groups of two areas separated by distance *d3* = 250 nm can be resolved using conventional microscopy, but areas within each group separated by distance *d1* = 50 nm remain unresolvable even without noise addition (Fig. 1c,d).

The srSESF images are presented in Fig. 1g–j as a map of correlation coefficients between the axial spatial periods profile at a given pixel and profiles at all other pixels versus lateral coordinate. In contrast to conventional microscopy, using the same objective lens with the resolution limit 400 nm, the fine lateral sample structure within groups, areas of 50 nm size separated just by 50 nm, are resolved using the srSESF approach (Fig. 1g,h). Utilizing information about internal structure of the sample, axial spatial period profiles for each image point, instead of just one intensity value as in conventional microscopy, also permits us to dramatically suppress the noise. Even after noise addition, at signal-to noise ratio (SNR) 45 dB, the areas within groups can be clearly distinguished (Fig. 1i,j).

**Figure 2:**
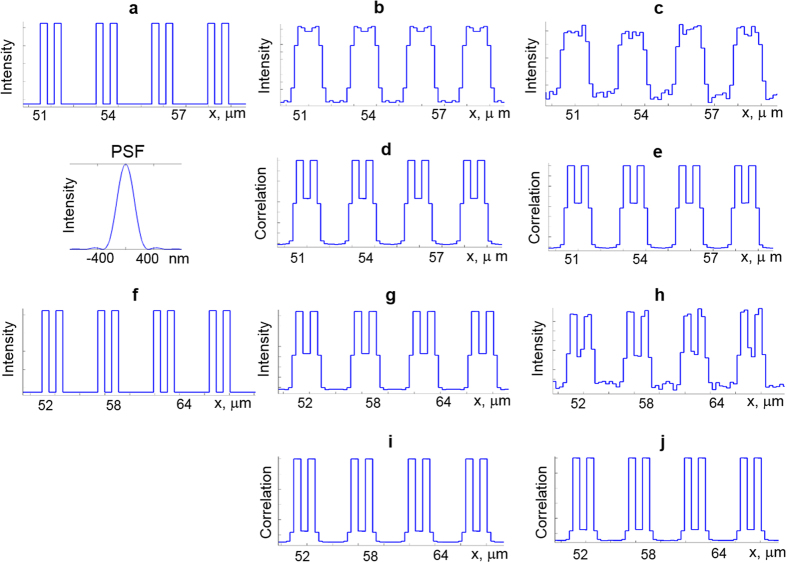
Results of numerical simulation. (**a**–**e**) for *d1* = 0.31 μm, *d2* = 0.31 μm, *d2* = 1.55 μm and (**f–j**) for *d1* = 0.56 μm, *d2* = 0.56 μm, *d2* = 2.8 μm. (**a**,**f**)–Lateral intensity distributions of the reflected light from the object. (**b**,**c**,**g**,**h**) Intensity distributions in the image plane using conventional microscopy. (**d**,**e**,**i**,**j**) Correlation coefficient distributions in the image plane using srSESF approach. (**b**,**d**,**g**,**i**)– without noise; (**c**,**e**,**h**,**j**)—with noise. PSF—point spread function for objective lens with NA = 0.9, wavelength 600 nm.

If the sizes of the lateral structures are increased (Fig. 2a), then it becomes possible not just to resolve two small features, but to accurately reconstruct the fine profile of the initial intensity on the sample using srSESF approach. We can see that conventional microscopy resolves the fine structure in the absence of noise (Fig. 2b), but this structure cannot be resolved after noise addition (Fig. 2c). The srSESF microscopy clearly resolves the fine structure of the sample (Fig. 2d), providing much better image contrast, and accurately reconstructs the profile of the initial intensity from the sample. Even in presence of noise, the srSESF approach accurately reconstructs the fine structure of the sample (Fig. 2e). If the sizes of the lateral structure are increased further (Fig. 2f), srSESF microscopy reconstructs the profile of the initial intensity from the object more accurately, as can be seen from comparison of the reconstructed using srSESF microscopy intensity profiles before noise addition Fig. 2i, and with noise Fig. 2j, and initial intensity profile Fig. 2f.

### Experiments

**Figure 3:**
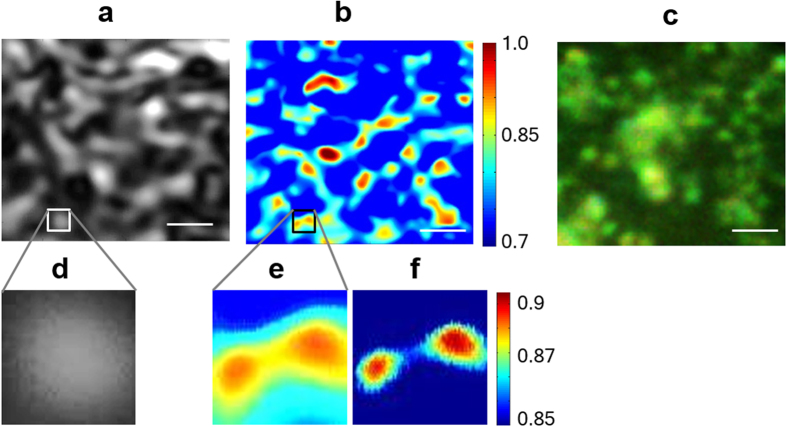
Images of the nanosphere aggregates: (**a**) scanning microscopy and (**b**) srSESF microscopy. Images (**a**,**b**) were formed using the wavelength range 1230 nm–1370 nm, NA = 0.5. Size of magnified portions in the images (**a**,**b**) is 1000 nm × 1000 nm. (**c**) Conventional bright field image using visible light, NA = 0.9. Scale bar is 2 microns.

Conventional scanning microscopy and srSESF images of the sample, made of nanospheres with 400 nm diameters, are presented in Fig. 3a and Fig. 3b correspondingly. Both conventional scanning microscopy and srSESF images were formed for the same area of the sample using an objective lens with NA = 0.5.

Improvement in resolution in image Fig. 3b in comparison with image Fig. 3a can be clearly seen. In magnified portions of the image in Fig. 3b two spheres can be seen separately, but in the corresponding portion of the conventional image Fig. 3a these spheres are totally indistinguishable. The contrast of the srSESF image can be changed by selection of different ranges of the correlation coefficients to better visualize the fine local structure, as demonstrated on the right magnified portion of the image in Fig. 3b. For reference, a conventional high resolution bright field image of the sample, formed in reflection configuration in the visible wavelength range, is also shown in Fig. 3c.

The experimental results show that the srSESF approach provides significant improvement in resolution. The resolution obtained is 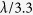 which is about 3 times better than diffraction limit of the imaging system used and even 1.3 times better than ultimate diffraction resolution limit for central wavelength 1300 nm.

**Figure 4:**
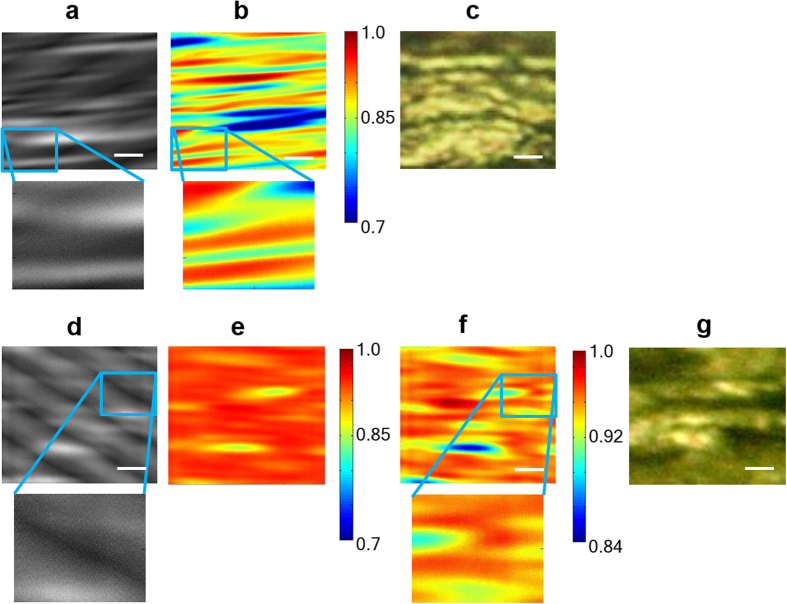
Images of collagen fibres: (**a**) and (**d**), scanning microscopy images with interference fringe noise; (**b**), (**e**) and (**f**), srSESF microscopy images formed using the wavelength range 1230 nm – 1370 nm, NA = 0.5, reveal the horizontal fibers; (**c**) and (**g**) high resolution conventional bright field images using visible light, NA = 0.8. The scale bar is 2 microns.

As an example of the application of the srSESF approach to biomedical objects, images of two different collagen tissues (Fig. 4a–g) are presented, where the two investigated tissues are obtained with (Fig. 4a–c) or without (Fig. 4d–g) dynamic stimulation, resulting in a different degree of fibre orientation (see details in METHODS for the tissue preparation). Figure 4a,d are scanning microscopy images, formed using objective lens with NA = 0.5, which are dominated by interference noise and do not clearly image the fibre with the exact orientation. The srSESF image Fig. 4b was formed

for the same area as the conventional scanning microscopy image. Figure 4a and the srSESF images Fig. 4e,f were formed for the same area as conventional scanning microscopy image Fig. 4d. The srSESF images in Fig. 4b,e were formed for the same correlation range. The srSESF image in Fig. 4f was formed with reduced correlation range. Figure 4c,g are conventional high resolution bright field images in reflection configuration using visible light.

The improvement in resolution is obvious if we compare conventional and srSESF images. Even from comparison between srSESF images Fig. 4b,e and the high resolution conventional bright field images (Fig. 4c,g) we can much better appreciate the structural heterogeneity, especially along the fibre. The correlation coefficients for most image points are increased and range of correlation reduced. Another important advantage of the proposed new contrast mechanism, as it was also demonstrated above using numerical simulation, is the ability to reduce noise and remove image artifacts. For example, interference noise which can be seen in conventional images Fig. 4a,d causing image artifacts such as apparent mis-orientation of the fibre (particularly evident in Fig. 4d), was removed in srSESF images Fig. 4b,e,f.

## Discussion

Demonstrated dramatic improvement in resolution is possible because instead of detection of negligible changes in intensity profiles, like in conventional microscopy, which are totally lost after convolution with the PSF of the imaging system even without noise, we analyze the internal structure, the axial spatial period profiles. In Supplementary information (Supplementary Fig. S1 and S2) we provide results of analysis of the axial spatial period profiles for two close lateral areas with similar internal structures and for two areas at the same separation with different internal structures, in the image plane, after convolution with the PSF of the imaging system. We used the same optical imaging system which was used to form images in Figs 1 and 2. The results are presented with and without noise addition. The sizes of the sample lateral and axial structures used to form Fig. S1 and S2 are the same as for Figs 1 and 2 correspondingly. The results in Fig. S1 confirm that it is possible to clearly distinguish the difference in axial spatial period profiles for two areas, separated by just 25 nm in lateral direction, after convolution with PSF of imaging system (resolution limit 400 nm) and in presence of noise. The Fig. S2 demonstrates accurate reconstruction of the axial spatial period profiles when the size of the sample structure increased.

Numerical simulation shows that resolution better than 1/6 of central wavelength 600 nm at 45 dB SNR, for thickness of the object of about 1 micron, can be achieved using the srSESF approach. The demonstrated resolution is about 4 times better than the diffraction limit of the imaging system used and more than two-fold better than the ultimate diffraction resolution limit for visible light. The resolution depends on the difference in internal structures between two points in the object (just 30 nm in our case) and, for the given axial structures, can be further improved by increasing SNR, the wavelength range and the thickness of the object. There is no limitation on the theoretically attainable resolution. Generally the approach can be extended to a broad class of objects, including absorbing media.

In summary, we proposed and demonstrated a new contrast mechanism for far field label free super-resolution imaging, srSESF microscopy. Instead of conventional image formation as 2D intensity distribution, srSESF microscopy forms images as a result of comparison of the axial spatial frequency (period) profiles, reconstructed for each image point. The nano-sensitivity of these profiles to structural alterations provides dramatic improvement in resolution. Potentially the srSESF approach can be realized with high frame rate using, for example, snapshot image mapping spectrometer (IMS)^29^ or swept wavelength light source. Improvement of resolution of 4 times in the presence of noise by numerical simulation and of about 3 times experimentally, relative to diffraction limit of the imaging system used, has been shown.

## Methods

### Super-resolution SESF approach

All biomedical objects are three dimensional, including cell cultures, single cells and cell constituents, collagen, etc. Reflected or transmitted light is a result of interaction of Illumination light with the internal structure. In conventional microscopy each point of image is formed as a superposition of all light waves after interaction with the internal structure at corresponding object’s point. Conventional images are two-dimensional (2D) intensity distributions in the image plane where each image point corresponds to one intensity value. The resolution can be defined as the shortest distance between two image points that results in a specific level of contrast between them to be distinguished. Two features within the object, which are too close and cannot be resolved using conventional microscopy, and area between them, usually have different internal structures in the depth direction. The idea of the srSESF approach, presented here, is to use this additional information about internal structure to resolve features in the lateral direction. We show that it is possible to detect the difference between fine features within object, separated in lateral direction, via calculation a difference in the axial (in depth direction) spatial frequency (or periods) profiles at points we want to resolve and points between them.

If the lateral structure sizes are too small to be resolved, then light, diffracted on this lateral structure, cannot pass through the optical imaging system. However, in the srSESF approach information about this fine lateral structure is encoded into the axial structure. Indeed, if there are no differences in structure, then the sample is uniform and there are no features to resolve. The lateral spatial separation between features we want to resolve is separation between corresponding axial spatial frequency profiles. In turn, information about axial spatial frequency profiles is spectrally encoded and can be passed through the optical system as different wavelengths^26^,^27^. So, the high spatial frequency information of lateral structure will be passed through the optical system as a difference in axial structure at different lateral locations, and the fine lateral structure, unresolved by conventional microscopy, can be resolved.

Super-resolution images are formed as differences between corresponding axial spatial frequency profiles. Different methods can be used for comparison of the axial spatial frequency profiles. For example, the srSESF image can be formed as a map of correlation coefficients between axial spatial frequency profile at a given image point, or profile of the numerically synthesized structure, and profiles at all other points, etc.

In information theory the fundamental resolution limit is set by the information capacity of the detected signal^30^. The srSESF approach dramatically increases the information capacity. Indeed, for each image point, instead of just one intensity value as in conventional microscopy, we will have axial spatial frequency profiles with hundreds or even thousands points.

The srSESF approach is realized in reflection configuration which facilitates *in vivo* tissue imaging. It is known that in reflection configuration back scattered light provides information about high axial spatial frequency content of the object^26^,^27^,^31^. The corresponding dominant axial spatial periods of the structure which scatters light are about half the wavelength. It means that, whenever the srSESF approach is applied, even “thin” specimens with thickness of about a few wavelengths will produce axial spatial frequency profiles encoding nano-sensitivity to structural changes.

### Numerical simulation

A sample, which consists of two lateral areas with similar axial structure (the five reflectors with similar axial spatial periods for two lateral areas) and area between them with different axial structure (the five reflectors with axial spatial periods which are different from axial spatial periods for two lateral areas we want to resolve), was numerically constructed (Fig. 1a). Thickness of the sample is about 1.2 microns and the refractive index is *n* = 1.35 which is typical for biomedical objects (cells). The group of two lateral areas with the same axial structure (230 nm axial period) and lateral size *d1* each are separated by an area *d2* which has a different axial structure with 200 nm axial period. This group is repeated along the lateral direction and the distance between two groups is *d2*.

A broadband plane wave with spectral range 450–750 nm was simulated for illumination. Images were formed as lateral intensity distributions after convolution of the reflected light with the point spread function (PSF) of the numerically simulated imaging system with numerical aperture *NA* = 0.9, resolution limit 400 nm. Namely, the lateral profiles of intensity *I*(*x*) of the reflected light for each wavelength were calculated in the sample plane. These profiles were convolved with the point spread function (PSF) of the numerically simulated imaging system to form images: *I*_*im*_ =|*U*|^2^*⊗*|*h*|^2^, where 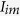 – intensity distribution in image plane, *U* – complex amplitude of the reflected light wave, *h*—PSF. The PSF was simulated as *h*(*r*) = 2*J*_1_(*ra*)/*ra*, where *J*_*1*_ is a Bessel function of the first kind. The value *a* is given by *a*=2*πNA*/*λ*, *NA*—numerical aperture and *λ*—wavelength. The example of a PSF for NA = 0.9 is presented in Fig. 2. Conventional images were formed as superposition of lateral intensity distributions for all wavelengths after convolution with PSF of the imaging system.

To form srSESF images the wavelengths were converted into the spatial periods according to the relation between wavelengths and spatial frequencies in K-space^27^,^31^. Profiles of axial spatial periods were reconstructed by taking the intensity at a given pixel for all lateral intensity distributions at all spatial periods. The srSESF images were formed as correlation maps between the axial spatial period profile (intensity versus spatial period) at the given pixel and axial spatial period profiles at all other pixels. An imaging spectrometer or swept light source can be used for recording the spectra. A linear array of detectors was simulated for detection. To simulate the real experimental situation, noise was added and SNR is 45 dB.

Numerical simulation was done for different sizes of the object structure: *d*_*1*_* = d*_*2*_ = 50 nm *d*_*3*_ = 250 nm, *d*_*1*_* = d*_*2*_ = 310 nm *d*_*3*_ = 1550 nm, and *d*_*1*_* = d*_*2*_ = 560 nm *d*_*3*_ = 2800 nm.

### Experiments

#### Scanning IR microscope

**Figure 5:**
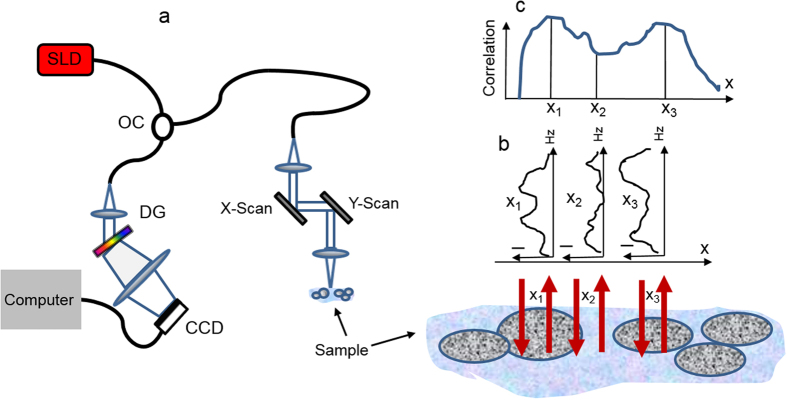
Schematic of the scanning microscope experimental setup with image acquisition. (**a**) – microscope, where SLD—superluminescent diode 1230 nm–1370 nm, OC—optical coupler, DG—diffraction grating. (**b**) – axial spatial period profiles for different lateral locations, (**c**) – srSESF image.

The scanning microscopy and srSESF images were acquired for the same areas of the samples using custom built scanning IR microscope (schematic is presented in Fig. 5). A broadband superluminescent diode light source, wavelength range 1230 nm–1370 nm, was used for illumination. The sample arm consisted of a pair of galvanometric driven mirrors and an objective lens with NA = 0.5. The spectrometer setup had a 1024 pixels InGaAs line scan camera (SU1024LDH2, Goodrich Ltd. USA) with a maximum acquisition rate of 91 kHz, spectral resolution 0.14 nm. This microscope is much simpler and cheaper than equipment usually used for super-resolution imaging.

### Samples

#### Phantom

To experimentally demonstrate improvement in resolution in comparison with conventional techniques the sample with nanosphere aggregates was made. The polymer spheres from Bangs Laboratories, Inc., diameter of spheres is 400 nm, were used to make the sample. An aliquot of about 10 μl of diluted monodispersed polystyrene nanosphere suspension (n = 1.59) was smeared uniformly onto a glass slide and dried, forming nanosphere aggregates.

#### Collagen scaffold tissue preparation

C2C12 cells (ATCC CRL-1772) were plated in Dulbecco’s modified Eagle’s medium (DMEM) supplemented with 10% fetal bovine serum (FBS), 100 units/ml penicillin, and 100 mg/ml streptomycin at 37 °C. Cells were grown to approximately 70–80% confluence and used for the experiments.

Collagen type I was extracted from rat tails and processed as previously described^32^. Briefly, 1 g of air-died and ultraviolet-sterilized collagen type I tendons extracted from rat tails were solubilized in 300 mL 0.1% acetic acid, obtaining a collagen acid solution at a concentration of 2 mg/ml, as quantified with BCA assay.

Collagen gels were then processed by mixing the sterile solution with a suspension of C2C12 murine myoblast (2*106). Collagen acidity was neutralized with NaOH (1 M) and NaHCO3 (0.26 M). This solution was then poured into a mold to obtain vessel-like scaffolds. After 24 h at 37 °C the collagen acid solution was jellified in the tube, with cells trapped within, and DMEM 10% FBS was placed as nutrient supplement for the cells (all from Lonza, Belgium).

Samples were kept in static condition for 21 days and then subjected where required to dynamic stimulation for 7 days in bioreactor using ElectroForce® BioDynamic™ Test Instruments. Assuming Poiseuille flow, the fluid speed used corresponds to a wall shear stress of 5 dyne/cm^2^. Pieces of collagen tissues, fixed in formaldehyde 4% water solution, were placed in a small dish and images were taken.

## Additional Information

**How to cite this article**: Alexandrov, S. A. *et al.* Novel approach for label free super-resolution imaging in far field. *Sci. Rep.* **5**, 13274; doi: 10.1038/srep13274 (2015).

## Supplementary Material

Supplementary Information
