# Supplementary material for: Novel approach for label free super-resolution imaging in far field
Source: Sci Rep. 2015 Sep 3;5:13274. doi: 10.1038/srep13274 (PMC4558609; doi:10.1038/srep13274)
Supplement: Supplementary Information [file srep13274-s1.pdf]

SUPPLEMENTARY INFORMATION

Novel approach for label free super-resolution imaging in far field

Sergey A. Alexandrov[1]*, James McGrath[1], Hrebesh Subhash[1], Francesca Boccafoschi[3], Cinzia Giannini[2] & Martin Leahy[1]

[1]Tissue Optics & Microcirculation Imaging Group, School of Physics, National University of Ireland, Galway, Ireland

[2]Institute of Crystallography, National Research Council, via Amendola 122/O, Bari 70126 Italy

[3] Department of Health Sciences, University of Piemonte Orientale "A. Avogadro", 28100 Novara, Italy

* Contact author: sergey.alexandrov@nuigalway.ie

Contents

Supplementary Figures 1, 2. Analysis of the axial spatial period profiles for two close areas of the sample with similar internal structure and for two areas with the same lateral separation with different internal structures, in the image plane, after convolution with the PSF of the imaging system and at presence of noise.

Supplementary Figure 1.

Supplementary Figure S1 shows that it is possible to detect the difference between two nearby pixels with different internal structures in the image plane (after convolution with PSF of the imaging system). The sizes of lateral areas are the same as in Fig. 1 in the manuscript, pixel size is 25 nm and so the distance between pixel centres is also 25 nm. The spatial period profiles at two nearby pixels with similar structure in the image plane (after convolution with PSF) remains the same (Supplementary Fig. S1b-e), whereas profiles at two nearby pixels

with different structure (Supplementary Fig. S1f) are different in the image plane (after convolution with PSF) (Supplementary Fig. S1g-j) even in presence of noise.

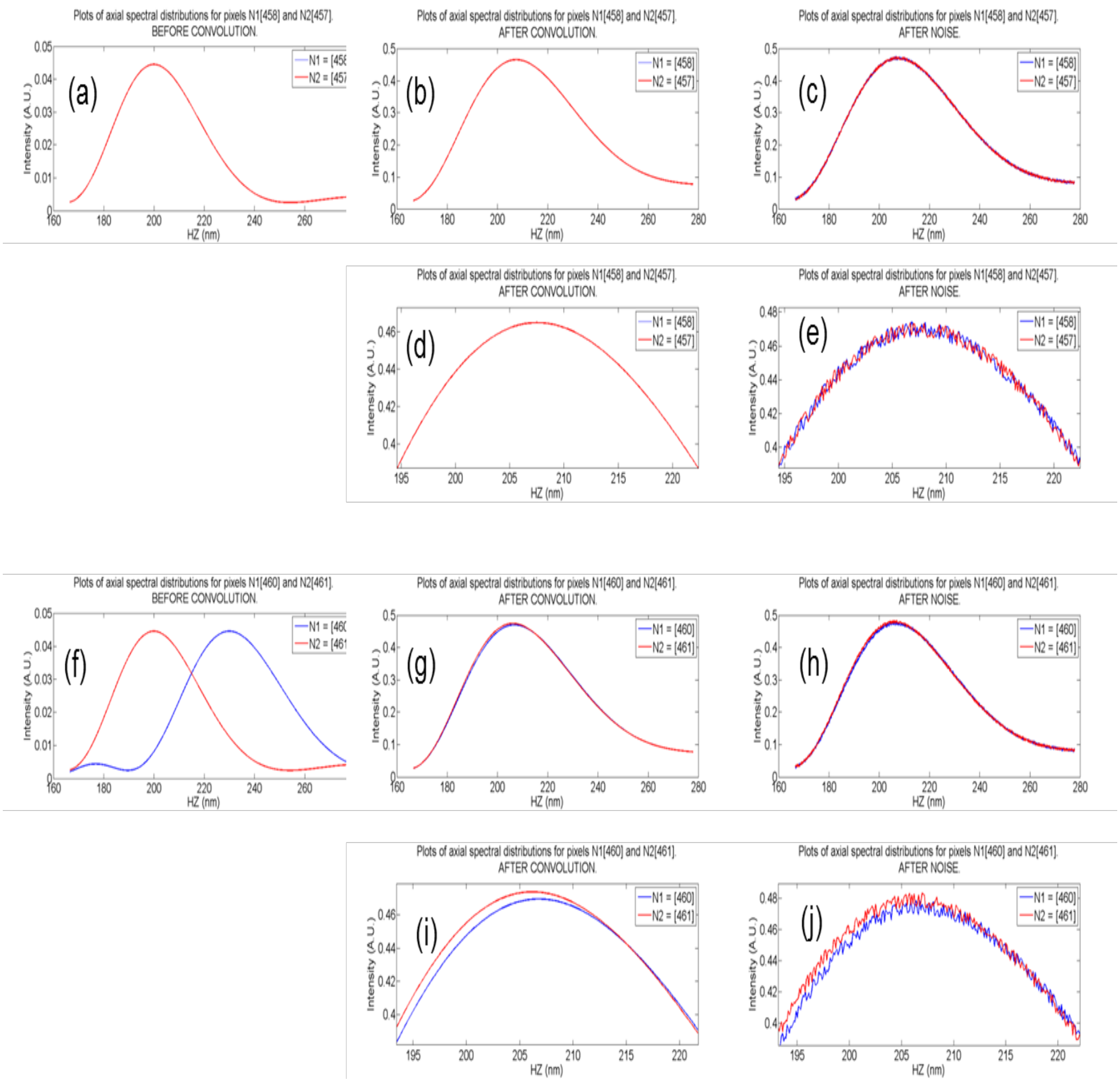

Supplementary Figure S1. Axial spatial period profiles (a) – (e) for two object pixels with similar internal structure and (f) – (j) for two object pixels with different internal structure. d_1= 50 nm, d_2 = 50 nm, d_3 = 250 nm. Profiles (a), (f) are in the object (before convolution with PSF), (b) – (e) and (g) – (j) - in the image (after convolution with PSF). Profiles (b), (g) are without noise and profiles (c), (h) - after noise addition. (d), (e) and (i), (j) are magnified portions of (b), (c) and (g), (h) correspondingly.

Supplementary Figure S2.

Supplementary Figure S2 shows axial spatial period profiles for two objects with increased sizes of lateral areas. The sizes of lateral areas and periods of internal structure are the same

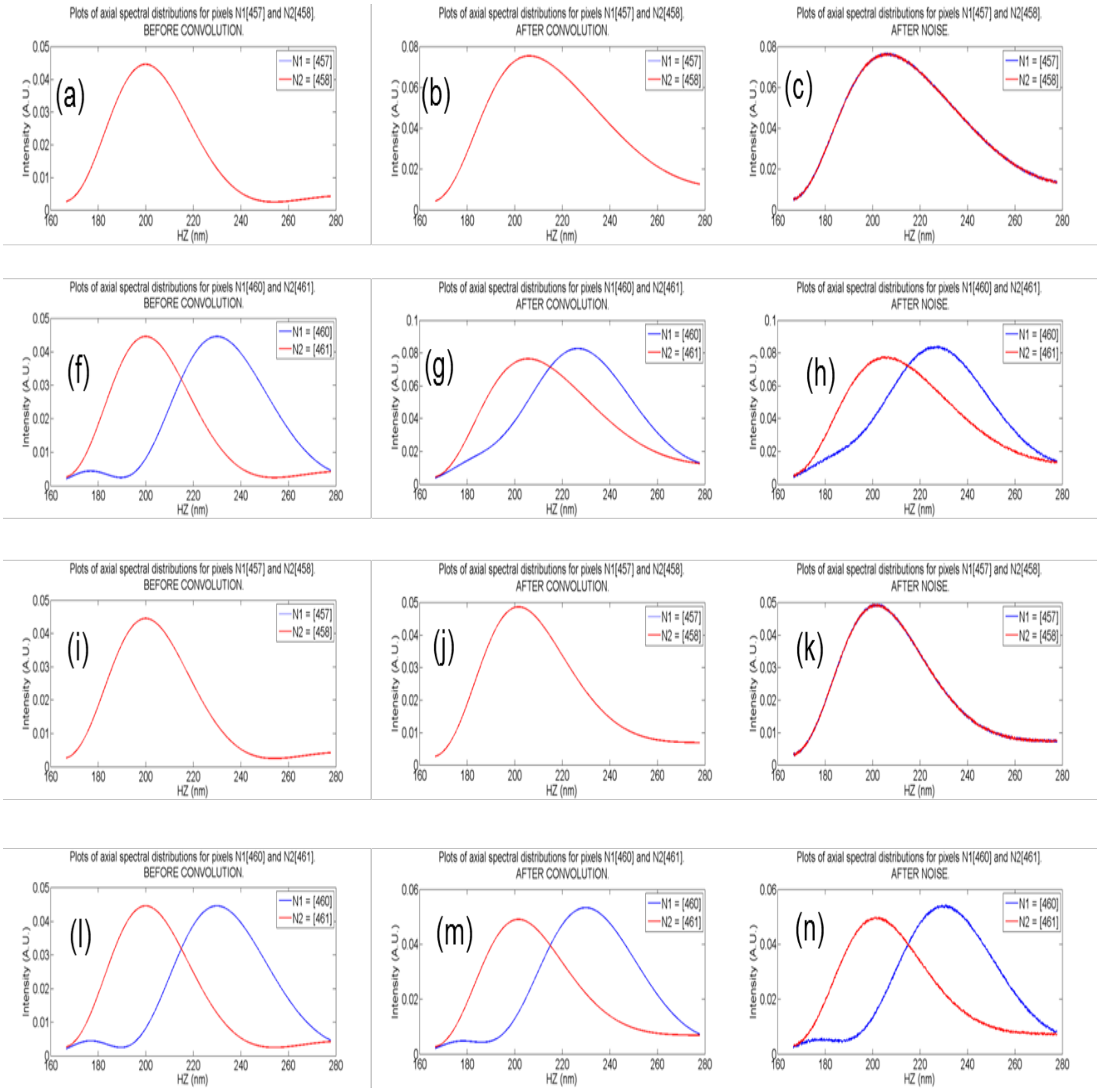

Supplementary Figure S2. Axial spatial period profiles. (a) – (h) for d_1= 0.31 µm, d_2 = 0.31 µm, d_3 = 1.55 µm. (i) – (n) for d_1 = 0.56 µm, d_2 = 0.56 µm, d_3 = 2.8 µm. (a) – (c) and (i) - (k) for two object pixels with similar internal structure and (f) – (h) and (l) – (n) for two object pixels with different internal structure. Profiles (a), (f), (i), (l) are in the object (before convolution with PSF), other profiles – in the image (after convolution with PSF). Profiles (b), (g), (j), (m) are without noise and profiles (c), (h), (k), (n) - after noise addition.

as in Fig. 2 in the manuscript. The Supplementary Fig. S2 clearly suggests that the axial spatial period profiles at two nearby pixels with similar internal structure in the image plane (after convolution with PSF) remains the same (Supplementary Fig. S2b,c and j,k) and are different for pixels with different internal structure (Supplementary Fig. S2g,h and m,n) even in presence of noise. When the size of lateral structure was increased, the initial axial spatial period profiles (Supplementary Fig. S2a,f,i,l) reconstructed more accurately (Supplementary Fig. S2j,k,m,n).
